# Dietary fats and their sources in association with the risk of bladder cancer: A pooled analysis of 11 prospective cohort studies

**DOI:** 10.1002/ijc.33970

**Published:** 2022-02-25

**Authors:** Mostafa Dianatinasab, Anke Wesselius, Amin Salehi‐Abargouei, Evan Y. W. Yu, Mohammad Fararouei, Maree Brinkman, Piet van den Brandt, Emily White, Elisabete Weiderpass, Florence Le Calvez‐Kelm, Marc J. Gunter, Inge Huybrechts, Maurice P. Zeegers

**Affiliations:** ^1^ Department of Complex Genetics and Epidemiology, School of Nutrition and Translational Research in Metabolism Maastricht University Maastricht The Netherlands; ^2^ Nutrition and Food Security Research Center, Department of Nutrition, School of Public Health Shahid Sadoughi University of Medical Sciences Yazd Iran; ^3^ Department of Epidemiology Shiraz University of Medical Sciences Shiraz Iran; ^4^ Department of Clinical Studies and Nutritional Epidemiology Nutrition Biomed Research Institute Melbourne Australia; ^5^ Cancer Epidemiology Division Cancer Council Victoria Melbourne Victoria Australia; ^6^ Department of Epidemiology, Schools for Oncology and Developmental Biology and Public Health and Primary Care Maastricht University Medical Centre Maastricht The Netherlands; ^7^ Fred Hutchinson Cancer Research Center Seattle Washington USA; ^8^ International Agency for Research on Cancer World Health Organization Lyon France; ^9^ CAPHRI School for Public Health and Primary Care Maastricht University Maastricht The Netherlands; ^10^ School of Cancer Sciences University of Birmingham Birmingham UK

**Keywords:** bladder cancer, diet, epidemiology, fat, oil, risk factor

## Abstract

The effects of fat intake from different dietary sources on bladder cancer (BC) risk remains unidentified. Therefore, the present study aimed to investigate the association between fat intakes and BC risk by merging world data on this topic. Data from 11 cohort studies in the BLadder cancer Epidemiology and Nutritional Determinants (BLEND) study, provided sufficient information on fat intake for a total of 2731 BC cases and 544 452 noncases, which yielded 5 400 168 person‐years of follow‐up. Hazard ratios (HRs), with corresponding 95% confidence intervals (CIs), were estimated using Cox‐regression models stratified on cohort. Analyses were adjusted for total energy intake in kilocalories, gender, smoking status (model‐1) and additionally for sugar and sugar products, beers, wine, dressing and plant‐based and fruits intakes (model‐2). Among women, an inverse association was observed between mono‐unsaturated fatty acids (MUFAs) and BC risk (HR comparing the highest with the lowest tertile: 0.73, 95% CI: 0.58‐0.93, *P*‐trend = .01). Overall, this preventative effect of MUFAs on BC risk was only observed for the nonmuscle invasive bladder cancer (NMIBC) subtype (HR: 0.69, 95% CI: 0.53‐0.91, *P*‐trend = .004). Among men, a higher intake of total cholesterol was associated with an increased BC risk (HR: 1.37, 95% CI: 1.16‐1.61, *P*‐trend = .01). No other significant associations were observed. This large prospective study adds new insights into the role of fat and oils in BC carcinogenesis, showing an inverse association between consumption of MUFAs and the development of BC among women and a direct association between higher intakes of dietary cholesterol and BC risk among men.

## Abstract 2

What's new?

The association between bladder cancer risk and fat intake from different dietary sources remains inconclusive. Here, Dianatinasab et al. investigated associations using pooled data from prospective 11 cohort studies in the Bladder Cancer Epidemiology and Nutritional Determinants consortium. Analyses show that while higher intake of monounsaturated fatty acids and plant‐based oils was associated with decreased bladder cancer risk, greater intake of cholesterol and animals fats was associated with increased risk, particularly in men. The findings offer insight into the role of fats and oils in the development of bladder cancer and lay the foundation for new dietary strategies in bladder cancer prevention.

**Abbreviations**
BC: bladder cancer
BLEND: Bladder cancer Epidemiology and Nutritional Determinants
BMI: body mass index
CI: confidence interval
EPIC: European Prospective Investigation into Cancer
FFQ: food frequency questionnaire
HRs: hazard ratios
MIBC: muscle‐invasive bladder cancer
MUFAs: mono‐unsaturated fatty acids
NLCS: The Netherlands Cohort Study
NMIBC: nonmuscle‐invasive bladder cancer
PUFAs: poly‐unsaturated fatty acids
RR: relative risk
SD: standard deviation
SFAs: saturated fatty acids
VITAL: VITamins and Lifestyle study

## 1 INTRODUCTION

According to the GLOBOCAN cancer statistics in 2020, bladder cancer is the 10th most commonly diagnosed cancer worldwide, with approximately 573 000 new cases and 213 000 deaths.^1^ Approximately 75% of BC cases are nonmuscle invasive bladder cancer (NMIBC) characterized by frequent recurrences, which requires intensive treatments and follow‐up measures, posing a large burden on the national health care budgets and patient's quality of life.^2^

Several epidemiological studies have identified factors that potentially influence BC risk. These factors include gender, smoking, age and occupation.^2^, ^3^ In addition, evidence suggests that factors related to lifestyle, physical activity and diet, might also affect the risk of BC.^4^, ^5^ Previous research on diet and BC reported that higher intakes of fluid, fruit, vegetables and yoghurt are associated with a reduced risk of BC.^6^

In addition, several dietary patterns have been associated with BC risk,^7^, ^8^ including a Western diet, which was shown to be associated with a higher BC risk,^9^, ^10^ and the Mediterranean diet, which was shown to be inversely associated with BC risk.^10^, ^11^ One of the major differences between the Western and the Mediterranean diet is the source of dietary fat.^12^ Accordingly, while the Mediterranean dietary fat intake mainly derives from plants such as olives (high in monounsaturated fats), the dietary fat intake from the Western diet mainly derives from animal products (high in saturated fats).^13^ This important difference may suggest that the sources of dietary fat might have different effects on BC risk. For example, in vitro studies on the effect of PUFAs on cancer may from anticarcinogenic to carcinogenic. PUFAs mainly found in vegetable oils (arachidonic acid) and meat (linoleic acid) are thought to induce tumor growth, by causing loss of cell viability and apoptosis,^14^, ^15^ while PUFAs are mainly found in ruminant meat (conjugated linoleic acid) and fat cold‐water fish (docosahexaenoic acids) are thought to reduce tumor growth, by reducing cell proliferation.^16^

Epidemiological evidence on the relation between dietary fat and BC and the various effects of different dietary fat sources, however, is scarce and inconclusive. While a Spanish case‐control study found that the observed increased BC risk with high intake of monounsaturated fatty acids (MUFAs) disappeared after adjustment for saturated fat,^17^ a Japanese case‐control study reported an inverse association between both saturated and monounsaturated fat intakes and BC risk.^18^ In addition, an observational study from Serbia highlighted the importance of the fat sources when establishing the effect of dietary fat intake on BC.^19^ The authors reported an inverse association of sunflower oil and BC risk, while a positive association was observed for animal fat intake.

Due to this current lack of knowledge and contradictory evidence, the present study aims to investigate the association between dietary fat intake from major sources and BC risk by pooling data from 11 prospective cohort studies.

## 2 METHODS

### 2.1 Study sample

The study was conducted within the Bladder Cancer Epidemiology and Nutritional Determinants (BLEND) consortium.^20^ BLEND is one of the largest international nutritional consortium, which includes 16 prospective cohort studies from 13 countries. For the present study, 11 cohort studies originated from 11 different countries (ie, Europe: European Prospective Investigation into Cancer and Nutrition cohort studies [EPIC]^21^ [Denmark, France, Germany, Italy, The Netherlands, Norway, Spain, Sweden, United Kingdom], Netherlands cohort study [NLCS]^22^ and North America: VITamins and Lifestyle cohort study [VITAL]^23^), with sufficient information on fat and oils consumption were eligible for inclusion in the present study.^20^

### 2.2 Data collection and coding

Details of the BLEND consortium protocol and methodology have been provided elsewhere.^20^ All included studies used a self‐administered or interview administered food frequency questionnaire (FFQ) that was validated on either food groups,^23^, ^24^, ^25^, ^26^, ^27^ and/or energy intake.^24^, ^27^, ^28^ The collected dietary fat intake was harmonized and categorized by using the hierarchal Eurocode 2 food coding system developed by the European Union.^29^ National specific standard portions sizes for each food item were used to calculate intake in gr/day. As a result of data availability, groups of fat and oils intakes were calculated in grams per day per 1000 kcal (g/1000 kcal/day, nutrient density method) to account for total energy intake and reduce extraneous variation in dietary intakes.^30^, ^31^ All fat and oils intakes were energy‐adjusted using the nutrient density method (in g/1000 kcal/day) and were categorized into tertiles for individual fat types.^31^ Dietary fats were classified as total lipids, total fatty acids, saturated fatty acids (SFAs), MUFAs, polyunsaturated fatty acids (PUFAs) and cholesterol. Also, dietary fat sources included: total fats and oils, plant‐based fats and oils, animal fat, cream, butter, margarine, corn oil, soya bean oil, rapeseed oil, grape seed oil, peanut oil, sunflower oil and olive oil in g/1000 kcal/day.

Person‐years of follow‐up for each participant was calculated from date of study enrolment until the date of BC diagnosis, or date of ending follow up (eg, date of death, lost to follow‐up or study exit), whichever came first.

Each study ascertained incident bladder cancer, defined to include all urinary bladder neoplasms according to the International Classification of Diseases for Oncology (ICD‐O‐3 code C67) using population‐based cancer registries, health insurance records or medical records.^32^ BCs were classified as nonmuscle invasive bladder cancer (NMIBC) or muscle invasive bladder cancer (MIBC). For the present study, the primary outcome was coded as BC cases or non‐BC cases, and the secondary outcome was coded as NMIBC, MIBC or non‐BC cases. NMIBC included noninvasive papillary carcinomas confined to the urothelium (stage Ta), and carcinomas that invaded the lamina propria of the bladder wall (stage T1). High grade flat noninvasive carcinomas confined to the urothelium (carcinoma in situ; CIS) without other concomitant tumor stages (ie, T1/Ta [classified to nonmuscle invasive prior] or muscle invasive) were also classified as NMIBC. MIBC included carcinomas that invaded into the detrusor muscle (stage T2), carcinomas that invaded into the perivesical tissue (stage T3) and carcinomas that invaded adjacent tissues and organs (most often the prostate or uterus, stage T4). In addition, nonmuscle‐invasive tumors were classified as NMIBC and muscle‐invasive as MIBC, if no further details were given on tumor stage and muscle‐invasive as MIBC.

In addition, to the information on dietary intake, the BLEND dataset also includes data on study characteristics (eg, design, method of dietary assessment, recall period of dietary intake), geographical region, demographic information (age, gender and ethnicity) and smoking (current/former/never) and its quantity (packs/year), which were measured at the baseline.

### 2.3 Statistical analysis

Baseline characteristics of the study participants, types of fat and oils and their dietary sources and other potential confounders were compared between case and noncase groups using analysis of variance or independent samples *t*‐test for continuous variables or *χ* ^2^ or ANCOVA for categorical variables.

To assess the influence of the different sources of dietary fat and BC risk, Cox proportional hazard regression was used to obtain hazard ratios (HRs) and corresponding 95% confidence intervals (CIs). Based on the adjusted model 2, the *P* for heterogeneity was calculated using the Wald test. The proportional hazards assumptions were examined graphically^33^ and no violation was observed.

Dietary fat intake was divided into three groups based on a tertile ordered distribution: low intake (tertile 1), medium intake (tertile 2) and high intake (tertile 3). The intake of some plant‐based fat sources was not variable enough to be categorized into tertiles (ie, corn oil, soya bean oil, rapeseed oil, grapeseed oil, peanut oil and sunflower oil). For these sources we used the median intake as a cut‐off to categorize the participants into low and high intake groups.

In the Cox regression model age was used as a time scale, thereby correcting for age in the analysis. Also, the effect of each study was analyzed as a random effect. The Cox regression models were fitted as crude, and adjusted models (adjusted for total energy intake in kilocalories [continuous], gender [women, men], smoking status [never, former or current smoker] [model 1] and additionally for: sugar and sugar products [continuous], beers [continuous], wine [continuous], dressing [continuous] and plant‐based and fruits intakes [continuous] [model‐2]). The analyses were stratified on gender and disease category (NMIBC and MIBC). To understand the relevance of interaction, the main interaction terms between fat and oils consumption and gender and smoking were added to the model 1. *P* values for trend were estimated by assigning medians to each category of consumption as a continuous variable.

Finally, in order to determine the study effect, sensitivity analysis was performed by removing each individual study from the main model. All statistical analyses were performed using Stata/SE version 14.2. *P*‐values less than .05 were considered as statistically significant.

## 3 RESULTS

### 3.1 Baseline characteristics

Baseline characteristics of the study population are presented in Table 1. Altogether, 2731 (2006 men and 725 women) cases and 544 452 (152 620 men and 391 832 women) noncases with a total of 5 400 168 person‐years of follow‐up (median follow‐up = 11.4 years) were included in our analysis. Compared to noncases, BC cases were older at baseline (mean = 60.4 years [SD: ±7.8] vs mean = 51.2 years [SD: ±10.6])) and were more likely to be male (73% vs 28%). Cases were mainly current (39%) or former smokers (42%), while noncases were more likely to be never smokers (50%). The mean intake of all fat types, including total lipid (34.4 vs 32.8 [g/1000 kcal/day]), total fatty acids (30.9 vs 29.5), SFAs (9.8 vs 9.1), MUFAs (12.5 vs 12.3), PUFAs (8.6 vs 8.0) and total cholesterol (128.7 vs 123.4) was statistically significantly higher among BC cases compared to noncases. The intakes of total fats and oils ([g/1000 kcal/day] 22.24 [14.01] vs 19.40 [12.90]), animal fat (0.22 [0.93] vs 0.16 [0.68]), butter (5.41 [11.27] vs 3.94 [8.26]), margarine (12.67 [14.89] vs 9.65 [12.60]) and rapeseed oil (0.04 [0.49] vs 0.02 [0.28]) were significantly higher among BC cases compared to noncases, while the intake of peanut oil was significantly higher among noncases (0.17 [1.09] vs 0.05 [0.57]). Additional baseline characteristics are provided in Table S1.

**TABLE 1:** Baseline characteristics and fat sources among noncases and bladder cancer cases in the BLEND international study

	Cases	Noncases
Categories of data	n = 2731	n = 544 452
Baseline age year (mean [SD])	60.36 (7.81)	51.16 (10.56)
Person‐year	*Total*: 20 784.46	*Total*: 5 379 384
	*Median*: 7.32	*Median*: 11.32
Gender, n (%)		
Men	2006 (73.45)	152 620 (28.03)
Women	725 (26.55)	391 832 (71.97)
Smoking status, n (%)		
Current	1057 (38.70)	111 967 (20.57)
Former	1142 (41.82)	164 637 (30.24)
Never	532 (19.48)	267 848 (49.20)
Fat and oil types, g/1000 kcal/day (mean [SD])		
Total lipid	34.41 (12.42)	32.76 (11.33)
Total Fatty acids	30.98 (1404)	29.47 (13.21)
Saturated fatty acids	9.78 (4.04)	9.15 (3.87)
Mono‐unsaturated fatty acids	12.56 (5.77)	12.27 (5.44)
Poly‐unsaturated fatty acids	8.64 (4.22)	8.05 (3.87)
Total cholesterol	128.75 (67.46)	123.42 (69.38)
Dietary fat sources, g/1000 kcal/day (mean [SD])		
Total fats and oils	22.24 (14.01)	19.40 (12.90)
Plant‐based fats and oils	5.72 (10.90)	5.53 (5.50)
Animal fat	0.22 (0.93)	0.16 (0.68)
Cream	2.44 (5.39)	2.29 (4.70)
Butter	5.41 (11.27)	3.94 (8.26)
Margarine	12.67 (14.89)	9.65 (12.60)
Corn oil	0.15 (0.89)	0.15 (1.38)
Soya bean oil	0.18 (1.14)	0.18 (1.07)
Rapeseed oil	0.04 (0.49)	0.02 (0.28)
Grape seed oil	0.04 (0. 54)	0.02 (0.39)
Peanut oil	0.05 (0.57)	0.17 (1.09)
Sunflower oil	0.62 (3.95)	0.71 (3.25)
Olive oil	4.49 (9.74)	4.18 (9.21)
Potential confounders		
Energy intake, kcal/day (mean (SD)	2161.03 (673.04)	2061.10 (633.82)
Fruits, g/1000 kcal/day (mean (SD))	115.47 (105.42)	115.55 (104.19)
Vegetables, g/1000 kcal/day (mean (SD))	203.70 (140.18)	182.97 (124.82)
Red and processed meat, g/1000 kcal/day (mean (SD))	636.03 (402.17)	505.50 (408.30)
Eggs, g/1000 kcal/day (mean (SD))	17.36 (15.21)	16.50 (15.68)
Sugar and sugar products, g/1000 kcal/day (mean (SD))	17.54 (22.37)	17.91 (44.41)
Beer, mL/day (mean (SD))	4.08 (9.53)	2.55 (7.32)
Wine, mL/day (mean (SD))	6.01 (13.50)	6.28 (12.03)
Dressing, g/1000 kcal/day (mean (SD))	4.78 (7.33)	6.18 (9.79)

Abbreviations: g, gram; kcal, kilocalorie; mg, milligram; mL, milliliters; SD, standard deviation.

### 3.2 Overall analysis

#### 3.2.1 Fat types and BC risk

The estimated HRs for the association between fat and oil intakes with BC are presented in Table 2. Overall, we found that higher consumption of MUFAs decreases the BC risk (HR_high vs low_: 0.84, 95% CI: 0.73‐0.96, *P*‐trend = .01), while higher intake of total cholesterol was associated with an increased BC risk (HR_high vs low_: 1.27, 95% CI: 1.05‐1.37, *P*‐trend = .01). No other fat types showed to be associated with BC risk.

**TABLE 2:** Hazard ratio (HR) and 95% confidence interval (CI) of the association of fat and oils types, and risk of BC based on tertile of fat and oils

	Tertile 1	Tertile 2	Tertile 3	
Fat and oil types	HR (95% CI)	HR (95% CI)	HR (95% CI)	*P* trend
*Total lipid*				
Participants (number)				
Case/noncase	430/139 575	454/139 551	541/139 464	—
Pearson year	1 523 368	1 543 497	1 611 960	—
Crude	1 (reference)	1.08 (0.96‐1.23)	1.04 (0.91‐1. 91)	.512
Model 1^a^	1 (reference)	1.14 (1.00‐1.30)	0.97 (0.85‐1.11)	.653
Model 2^b^	1 (reference)	1.13 (0.99‐1.29)	0.95 (0.83‐1.09)	.451
*Total fatty acids*				
Participants (number)				
Case/noncase	411/139 594	414/139 591	600/139 405	
Pearson year	1 539 499	1 527 401	1 611 925	—
Crude	1 (reference)	0.96 (0.84‐1.11)	1.16 (1.02‐1.31)	.015
Model 1^a^	1 (reference)	0.97 (0.84‐1.12)	0.94 (0.82‐1.06)	.342
Model 2^b^	1 (reference)	0.96 (0.84‐1.11)	0.90 (0.79‐1.03)	.161
*Saturated fatty acids*				
Participants (number)				
Case/noncase	376/139 629	463/139 542	586/139 419	—
Pearson year	1 523 593	1 565 893	1 589 340	—
Crude	1 (reference)	1.18 (1.03‐1.35)	1.23 (1.08‐1.41)	.002
Model 1^a^	1 (reference)	1.14 (0.99‐1.31)	1.09 (0.95‐1.25)	.235
Model 2^b^	1 (reference)	1.12 (0.97‐1.28)	1.04 (0.91‐1.20)	.583
*Mono‐unsaturated fatty acids*				
Participants (number)				
Case/noncase	485/139 520	448/139 557	492/139 513	—
Pearson year	1 526 609	1 546 260	1 605 956	—
Crude	1 (reference)	0.91 (0.80‐1.04)	0.82 (0.72‐0.94)	.004
Model 1^a^	1 (reference)	0.96 (0.84‐1.09)	0.83 (0.73‐0.95)	.008
Model 2^b^	1 (reference)	0.98 (0.86‐1.11)	0.84 (0.73‐0.96)	.013
*Poly‐unsaturated fatty acids*				
Participants (number)				
Case/noncase	426/139 579	434/139 571	565/139 440	—
Pearson year	1 516 905	1 535 911	1 626 010	—
Crude	1 (reference)	1.08 (0.94‐1.23)	1.23 (1.08‐1.40)	.001
Model 1^a^	1 (reference)	1.00 (0.88‐1.15)	0.97 (0.85‐1.10)	.611
Model 2^b^	1 (reference)	1.01 (0.88‐1.15)	0.96 (0.84‐1.10)	.604
*Total cholesterol*				
Participants (number)				
Case/noncase	409/139 596	482/139 523	534/139 471	—
Pearson year	1 582 561	1 545 622	1 550 642	—
Crude	1 (reference)	1.11 (0.97‐1.26)	1.17 (1.03‐1.33)	.006
Model 1^a^	1 (reference)	1.09 (0.96‐1.25)	1.17 (1.03‐1.34)	.031
Model 2^b^	1 (reference)	1.11 (0.97‐1.26)	1.27 (1.05‐1.37)	.017

Abbreviations: CI, confidence interval; HR, hazard ratio.

^a^ Adjusted for smoking status, age, gender and total energy intake in kilocalories.

^b^ Adjusted for model 1+ sugar and sugar products, beers, wine, dressing, vegetables and fruits.

#### 3.2.2 Fat sources and BC risk

High consumption of animal fats showed to be associated with an increased BC risk (HR_high vs low_: 3.76, 95% CI: 3.43‐4.12; *P*‐trend = .001), while an inverse association was observed between BC risk and high intake of both plant‐based oils and oils and sunflower oil (HR_high vs low_: 0.94, 95% CI: 0.82‐0.99; *P*‐trend = .09 and HR_high vs median_: 0.72, 95% CI: 0.58‐0.90; *P*‐trend = .004, respectively). No other fat sources showed to be associated with BC risk (Tables 3 and 4).

**TABLE 3:** Hazard ratio (HR) and 95% confidence interval (CI) of the association of fat and oils intake, and risk of BC based on tertile of fat and oils

	Tertile 1	Tertile 2	Tertile 3	
Fat and oil source	HR (95% CI)	HR (95% CI)	HR (95% CI)	*P* trend
*Total fats and oils*				
Participants (number)				
Case/noncase	367/139 638	478/139 527	580/139 425	—
Pearson year	1 515 977	1 536 487	1 626 361	—
Crude	1 (reference)	1.28 (1.12‐1.47)	1.39 (1.12‐1.58)	.001
Model 1^a^	1 (reference)	1.08 (0.93‐1.24)	1.05 (0.92‐1.21)	.476
Model 2^b^	1 (reference)	1.07 (0.93‐1.23)	1.04 (0.91‐1.19)	.574
*Plant‐based fats and oils*				
Participants (number)				
Case/noncase	617/139 388	374/139 631	434/139 571	
Pearson year	1 594 911	1 510 110	1 573 804	—
Crude	1 (reference)	0.79 (0.69‐0.90)	0.89 (0.78‐1.01)	.056
Model 1^a^	1 (reference)	0.95 (0.83‐1.08)	1.01 (0.97‐1.27)	.140
Model 2^b^	1 (reference)	0.94 (0.82‐1.07)	0.94 (0.82‐0.99)	.097
*Animal fat*				
Participants (number)				
Case/noncase	1330/395 682	95/22 680	208/125 853	
Pearson year	4 422 730	253 572.4	2522.467	—
Crude	1 (reference)	1.42 (1.15‐1.75)	3.17 (2.93‐3.44)	.001
Model 1^a^	1 (reference)	1.31 (1.06‐1.62)	4.82 (4.18‐5.52)	.001
Model 2^b^	1 (reference)	1.35 (1.09‐1.67)	3.76 (3.43‐4.12)	.001
*Butter*				
Participants (number)				
Case/noncase	654/139 351	355/139 650	416/139 589	
Pearson year	1 613 190	1 542 855	1 522 779	—
Crude	1 (reference)	0.78 (0.68‐0.89)	0.78 (0.69‐0.88)	.001
Model 1^a^	1 (reference)	0.98 (0.86‐1.12)	1.01 (0.89‐1.15)	.840
Model 2^b^	1 (reference)	0.92 (0.81‐1.05)	0.93 (0.82‐1.06)	.283
*Cream*				
Participants (number)				
Case/noncase	553/139 452	424/139 581	448/139 557	
Pearson year	1 581 301	1 516 669	1 580 854	—
Crude	1 (reference)	0.82 (0.72‐0.93)	0.81 (0.71‐0.92)	.001
Model 1^a^	1 (reference)	0.91 (0.79‐1.03)	0.96 (0.82‐1.10)	.584
Model 2^b^	1 (reference)	0.89 (0.80‐1.04)	0.91 (0.80‐1.04)	.172
*Margarine*				
Participants (number)				
Case/noncase	414/139 591	410/139 595	601/139 404	
Pearson year	1 545 415	1 521 002	1 612 407	—
Crude	1 (reference)	0.95 (0.83‐1.10)	1.15 (1.02‐1.31)	.015
Model 1^a^	1 (reference)	0.92 (0.80‐1.06)	0.92 (0.81‐1.05)	.275
Model 2^b^	1 (reference)	0.95 (0.82‐1.09)	0.90 (0.78‐1.03)	.136
*Olive oil*				
Participants (number)				
Case/noncase	781/139 683	211/86 335	433/139 572	
Pearson year	2 201 173	932 829	1 544 823	—
Crude	1 (reference)	0.89 (0.77‐1.04)	1.06 (0.94‐1.20)	.23
Model 1^a^	1 (reference)	1.02 (0.91‐1.14)	0.94 (0.84‐1.06)	.15
Model 2^b^	1 (reference)	1.10 (0.10‐1.24)	1.05 (0.90‐1.22)	.27

Abbreviations: CI, confidence interval; HR, hazard ratio.

^a^ Adjusted for model 1+ sugar and sugar products, beers, wine, dressing, vegetables and fruits.

^b^ Adjusted for smoking status, age, gender and total energy intake in kilocalories.

**TABLE 4:** Hazard ratio (HR) and 95% confidence interval (CI) of the association of different vegetable oils intake according to median of intakes, and risk of BC

Fat and oil source	Under median	Above median	*P* value
*Corn oil*			
Participants (number)			
Case/noncase	1334/400 459	81/18 131	—
Pearson year	4 462 137	216 687.9	—
Crude	1 (reference)	1.05 (0.84‐1.31)	.661
Model 1^a^	1 (reference)	1.10 (0.88‐1.39)	.374
Model 2^b^	1 (reference)	1.03 (0.82‐1.29)	.760
*Soya bean oil*			
Participants (number)			
Case/noncase	1294/362 613	131/55 977	—
Pearson year	4 057 981	620 844	—
Crude	1 (reference)	0.85 (0.71‐1.02)	.089
Model 1^a^	1 (reference)	1.02 (0.85‐1.23)	.784
Model 2^b^	1 (reference)	1.05 (0.87‐1.27)	.574
*Rapeseed oil*			
Participants (number)			
Case/noncase	1405/415 701	20/2889	—
Pearson year	4 646 888	31 937.39	—
Crude	1 (reference)	1.42 (0.91‐2.22)	.134
Model 1^a^	1 (reference)	0.98 (0.61‐1.56)	.940
Model 2^b^	1 (reference)	1.18 (0.76‐1.85)	.444
*Grape seed oil*			
Participants (number)			
Case/noncase	1408/415 777	19/2813	—
Pearson year	4 647 315	31 509.75	
Crude	1 (reference)	1.28 (0.79‐2.06)	.309
Model 1^a^	1 (reference)	1.01 (0.94‐1.64)	.948
Model 2^b^	1 (reference)	1.13 (0.70‐1.83)	.602
*Peanut oil*			
Participants (number)			
Case/noncase	1403/396 221	22/22 369	—
Pearson year	4 440 423	238 402.4	—
Crude	1 (reference)	0.30 (0.20‐0.47)	.001
Model 1^a^	1 (reference)	0.84 (0.52‐1.36)	.490
Model 2^b^	1 (reference)	0.66 (0.43‐1.02)	.063
*Sunflower oil*			
Participants (number)			
Case/noncase	1335/360 389	90/58 201	—
Pearson year	4 032 881	645 943.9	—
Crude	1 (reference)	0.45 (0.36‐0.56)	.001
Model 1^a^	1 (reference)	0.80 (0.64‐1.01)	.064
Model 2^b^	1 (reference)	0.72 (0.58‐0.90)	.004

Abbreviations: CI, confidence interval; HR, hazard ratio.

^a^ Adjusted for smoking status, age, gender and total energy intake in kilocalories.

^b^ Adjusted for model 1+ sugar and sugar products, beers, wine, dressing, vegetables and fruits.

### 3.3 Stratified analysis

#### 3.3.1 Fat types and BC risk by gender and BC subtypes stratification

Significant heterogeneity between men and women was observed in the associations of MUFAs and total cholesterol intake with BC (*P*‐het = .001 and <.001, respectively). Interestingly, higher intakes of MUFAs significantly decreased the risk of BC for women (HR_high vs low_: 0.73, 95% CI: 0.58‐0.93, *P* trend = .01), but not for men (HR_high vs low_: 0.94, 95% CI: 0.80‐1.11; *P*‐het = .001). In contrast, higher intakes of total cholesterol significantly increased the risk of BC for men (HR_high vs low_: 1.37, 95% CI: 1.16‐1.61; *P*‐trend = .001), but not for women (HR_high vs low_: 0.90, 95% CI: 0.71‐1.13, *P*‐het = .001). No other associations were found in neither men nor women (Table S2).

Higher intakes of total lipids significantly decreased the NMIBC risk (HR_high vs low_: 0.73, 95% CI: 0.55‐0.96; *P* trend = .01), but not the MIBC risk (HR_high vs low_: 1.19, 95% CI: 0.65‐1.17, *P*‐het = .001). Also, higher intakes of MUFAs significantly decreased the NMIBC risk (HR_high vs low_: 0.69, 95% CI: 0.53‐0.91, *P* trend = .004), but not the MIBC risk (HR_high vs low_: 0.86, 95% CI: 0.44‐1.64; *P*‐het = .002; Table S3).

#### 3.3.2 Fat sources and BC risk by gender and BC subtypes stratification

Higher intakes of total fat and oils, butter and margarine were found to significantly increase the risk of BC for men (HR_high vs low; fats and oils_: 1.34, 95% CI: 1.17‐1.53; HR _high vs low; butter_: 1.42, 95% CI: 1.27‐1.58; HR _high vs low; margarine_: 1.70, 95% CI: 1.50‐1.92), but not for women. No other sources showed to be associated with BC risk either for men nor women (data are not shown). Stratification by NMIBC and MIBC and fat sources shows relatively similar results to the overall findings.

No single study effect could be observed. After removing each individual study from the main model results remained the same.

## 4 DISCUSSION

To our best knowledge, this is the first pooled longitudinal cohort study on the associations between different types and sources of fat and oils and BC risk. Here we found that a high intake of MUFAs was significantly associated with a decreased risk in BC, particularly in women. In contrast, higher intake of cholesterol was associated with an increased BC risk, particularly in men. In addition, we found that higher consumption of animal fat was associated with an increased BC risk, while plant‐based fats and oils and sunflower oil decrease BC risk.

During the last decade the role of MUFA, primarily oleic acid (OA) (18:1n‐9), has attracted much attention. Especially since the Mediterranean diet, which is rich in olive oil (and thereby rich in MUFAs), has been traditionally linked to a protective effect on cancer,^34^ however, epidemiological evidence on the effect of MUFAs on BC risk, is scarce and inconclusive.^35^, ^36^, ^37^ The present study shows that high intake of MUFAs is associated with an overall decreased BC risk. When stratifying for BC subtype, results show that this association, only remained significant for the NMIBC subtype. However, data on BC subtypes was mainly lacking (ie, 8 out of 11 studies provided information on BC subtypes), resulting in low power, thereby hampered the statistical power to find a small effect size.

The positive associations found between MUFA intake and BC risk are in agreement with a recent meta‐analysis of observational studies and a Japanese case‐control study, also suggesting an inverse association between high intake of MUFAs and BC risk.^18^, ^38^ In contrast, two previously conducted cohort studies on MUFAs intake and BC risk reported a null association.^37^, ^39^ Moreover, a Spanish multicenter case‐control study found a slightly increased BC risk for high MUFA intake.^17^ Interestingly, however, this initially found positive association disappeared after adjustment for saturated fat intake. A possible explanation for these controversial findings might be the source of the MUFAs. Monounsaturated fat can be obtained from either olive oil^34^ or from animal sources, for example, beef,^12^ which showed to have an opposite effect on BC risk.^40^

In our study we observed an inverse association between plant‐based fats and oils intakes and BC risk. This is in line with findings of the New Hampshire case‐control study, also suggesting a decreased BC risk with high vegetable oil intake.^37^ In addition, Brinkman et al, reported a clear reduced BC risk for high intakes of α‐linolenic acid and vegetable fat. Furthermore, the same study showed a reduced BC risk was observed for polyunsaturated fat and linoleic acid.^37^ The protective effects of plant‐based oils, could be explained by its provision of various amounts of MUFAs, PUFAs and energy, which are potential antioxidants and chemopreventive factors that might affect the initiation, promotion and progression of cancers through several potential biologic mechanisms, including reduced cellular oxidative stress and probably decreased DNA damage.^41^

In the last two decades, there have been puzzling results regarding the possible role of dietary olive oil in cancer prevention and treatment.^42^ Oleic acid, which is a MUFA that is highly available in olive oil, canola oil, sunflower oil, soybeans oil, rapeseed oil and peanuts oil has been traditionally linked to a protective effect on cancer.^34^ It is, therefore, surprising that the present study shows no effect of olive oil (MUFA: 73% vs PUFAs: 11%) and nor rapeseed oils (MUFA: 62% vs PUFAs: 32%) intakes on BC risk. This null‐effect, however, has been observed in a previous study in which oils rich in MUFAs, derived from the seeds of soybean or grapeseed oil, did not exert health benefits and may not be associated with BC risk.^43^ This may be extrapolated to olive oils. Our results further indicate that the protective effect of MUFA on BC risk is explained by dietary intake of multiple sources.

Interestingly, stratification for gender showed that a high intake of MUFAs may significantly decrease the risk of BC for women but not for men. Wakai et al also reported gender discrepancy in the association of MUFAs intake and BC risk.^18^ This discrepancy might be related to overall gender differences in reporting diet^44^ this is because women may report their diet biased toward healthier habits and genetics, causing a different effect of similar environmental exposures to the bladder carcinogenesis.^45^ Furthermore, the sex hormone profile in itself (especially androgens) might play a role in the development and progression of BC.^46^ It should be taken into account, however, that the present study contains a limited number of female cases (n = 642), which could have led to a power issue, thereby enabling the detection of small size effects. However, it cannot be ruled out that residual confounding by other factors might explain the gender difference. Therefore, future research is needed to clarify this gender difference in the role of MUFA's on BC risk. Total lipid intake was shown to significantly decrease the NMIBC risk but not the MIBC risk. However, as mentioned before, data on BC subtype was mainly lacking, resulting in low power for detailed analysis. In our study it was shown that the total lipid intake was mainly derived from MUFA. Since MUFAs are suggested to have a protective role against BC, the association found between total lipid intakes and NMIBC might be related to higher intakes of MUFAs.

ω‐3 PUFAs have been reported as one potential modifiable protective factor against cancers.^47^ Although not fully understood, it is suggested that n‐3 PUFAs may possibly inhibit carcinogenesis through its anti‐inflammatory activity.^39^ Epidemiological studies on the intake of PUFAs and BC risk, however, showed inconsistent results. While some studies showed a null association between PUFA intake and BC risk,^17^, ^18^ others reported an inverse association.^35^ In the present study a null association was observed. For fat and oil sources, which contain more PUFAs than MUFAs, a similar noneffect was shown for soybean (MUFA: 24% vs PUFAs: 61%) and corn oil (MUFA: 24% vs UFA: 59%) and BC risk. However, when assessing sunflower oil (MUFA: 20%, vs PUFAs: 69%) independently, an inverse association with BC risk was observed. The controversial results obtained in different studies might again be due to the different sources (ie, animal and different plants) from which the PUFAs derive.^34^ Besides, also the cooking method of the PUFA sources might explain the variability in the results of the different studies.^39^

Limited evidence and contradictory findings are available on the association between a high trans fatty acids (TFAs) intake and BC risk.^37^, ^48^, ^49^ While several studies reported a direct association between higher TFAs intakes and BC risk,^48^, ^49^ others reported no significant association.^37^, ^50^ The present study also showed no evidence for an association between TFAs and BC risk, or for high intake of SFAs and PUFAs.

In recent years, cholesterol has received increasing attention due its role in carcinogenesis.^51^ Clinical and experimental evidence suggest that an increased cholesterol level in blood is associated with a higher cancer risk and that cholesterol‐lowering drugs (eg, statins) exhibit beneficial effects on bladder cancer development.^52^ So far, some mechanisms have been suggested to explain the possible role of cholesterol in the development of cancer, including; (a) changes in lipid and apolipoprotein levels that may result in cellular inflammation, by increasing the levels of proinflammatory cytokines, including tumor necrosis factor‐α and interleukin‐6^53^ and (b) the deregulation of cholesterol homeostasis through the disruption of the cholesterol pathway and the induction of elevated mitochondrial cholesterol levels leading to resistance to apoptotic signals.^52^ In the present study we found that cholesterol was associated with an increased BC risk among men but not among women. The null‐association observed among women is in line with results from a Belgian case‐control study and the New Hampshire case‐control, showing an overall null‐association between cholesterol intake and BC risk.^37^, ^39^ Since, evidence on the gender specific relations between cholesterol and BC risk are scarce, it remains unclear why in the present study a discrepancy between men and women was observed. However, the involvement of certain steroids, such as estrogen, in reducing the adverse effects of cholesterol, might explain the observed difference. Estrogen is proposed to protect against chronic diseases (ie, breast cancer and cardiovascular diseases or atherosclerosis) via its role in reverse cholesterol transport.^54^ Furthermore, increased use of statins among men compared to women need to be taken into account in future research on the gender specific relation between cholesterol and BC.

It is suggested that animal fat increases oxidative stress and levels of reactive oxygen species (ROS) that interfere with cellular processes. Healthy cells are attacked by free radicals, which cause peroxidation and eventually DNA damage. Thereby, ROS can lead to tumor initiation and progression of cancer cells.^55^ The present study strengthens this hypothesis by showing an increased BC risk associated with an animal fat intake, which is in line with a previously conducted case‐control study.^19^ However, Brinkman et al showed a null association between intakes of animal fat and BC risk.^37^ Again, this observed difference between the different studies might be due to the different type, composition and cooking method of the consumed animal fats included in the analysis.

No association was observed for higher intakes of total fats and oils and BC risk. Contrary to our finding, a meta‐analysis revealed that the total dietary fat intake increases the BC risk.^38^ However, this could only be observed among the European populations, while no association was reported for the North American populations.^38^

### 4.1 Strengths and limitations

In our knowledge, BLEND is one of the largest pooled cohort studies investigating the associations between the intake of different sources of fat and oils and risk of developing BC, thereby allowing to performed detailed analysis to find small effect sizes. Nevertheless, the present study has some limitations; (a) some of the dietary information was only available in portions per week. Though this data was converted to grams per day using the United States Department of Agriculture (USDA) food composition database, the conversions were not country specific. Previous studies, however, suggested that the application of a common food composition database has advantages over the use of country specific food composition databases in that errors are consistent between the countries, hence making data more comparable^56^; (b) unfortunately, data potential known BC risk factors, such as BMI, physical inactivity, socioeconomic status and occupational exposures to carcinogenic chemicals was missing. Moreover, it might be possibility that some lifestyle and/or environmental factor are associated with an individual's diet. Generally, people with a healthier diet have an overall healthier lifestyle. However, the current literature suggests only a small proportion of BC cases can be attributed to lifestyle and environmental factors^3^, ^57^; (c) although people are less likely to change their dietary habits at an older age, most of the included studies only measured their participants at baseline and we were, therefore, unable to take possible changes of dietary habits over time into account. This could have led to misclassification of long‐term exposure; (d) the effect of fat and oil on bladder carcinogenesis might be induced by compounds related to the cooking and processing of fat and oils. However, in the present study no information on fat and oils preparation or cooking methods was available, thereby lacking the ability to adjust or stratify on these factors; (e) for most cohorts the exposure and outcome variable was assessed by FFQs, therefore, measurement error and misclassification of study participants in terms of the exposure and outcome are unavoidable. Likewise, information bias, as a consequence of self‐reported information on food consumption, might have occurred. However, the strength and direction of this type of bias is not expected to be significantly different between cases and noncases, and therefore the impact of information bias is expected to be minimal; (f) although we found similar results after adjusting for potential dietary risk factors, it remains possible that the observed associations were confounded by other dietary constituents or additives associated with fat intake; (g) the present study sample consists mostly of Caucasians, and this may limit the generalizability of our results to other racial/ethnic populations or geographic regions; (h) although status as well as duration and intensity of smoking were taken into account in our analysis, the adjustment for smoking might still be imperfect due to differences in smoking practices (eg, depth of inhalation or amount of inhalation), differences in types of smoke exposure or lack of information on passive smoking^58^; (i) some tumor subtype information was missing, which hampered the statistical power required for stratified subgroup analyses.

## 5 CONCLUSIONS

In conclusion, this large prospective study adds new insights into the role of fat and oils in BC development. Results revealed that higher dietary cholesterol and animal fat intake might increase the BC risk in men, while higher intake of MUFAs and plant‐based oils decrease the BC risk in women. These findings suggest that BC prevention strategies should include a nutritional scheme that controls for the quality of fat consumed. However, further experimental, prospective and interventional studies are needed to clarify the exact effects and mechanisms of fat and oils in the etiology of BC.

## CONFLICT OF INTEREST

The authors declare that they have no conflict of interest.

## AUTHOR CONTRIBUTIONS

Mostafa Dianatinasab, Anke Wesselius, Maurice P. Zeegers and Maree Brinkman were involved in the study conceptualization, methodology, writing and editing the article. Amin Salehi‐Abargouei, Evan Y.W. Yu and Mohammad Fararouei helped in data analysis and article review. Piet van den Brandt, Emily White, Elisabete Weiderpass, Florence Le Calvez‐Kelm, Marc J. Gunter and Inge Huybrechts were involved in writing and editing the article and providing critical feedback. All authors have read and agreed to the published version of the article.

## ETHICS STATEMENT

Each participating study has been approved by the local ethic committee. Informed consent was obtained from all individual participants included in each study.

## Supporting information


**Appendix S1** Supporting Information.

## Data Availability

Datasets that are minimally required to replicate the outcomes of the study will be made available upon reasonable request.
