# Supplementary material for: Dietary fats and their sources in association with the risk of bladder cancer: A pooled analysis of 11 prospective cohort studies
Source: Int J Cancer. 2022 Feb 25;151(1):44–55. doi: 10.1002/ijc.33970 (PMC9303525; doi:10.1002/ijc.33970)
Supplement: Supplementary file 1 — Appendix S1 Supporting Information. [file IJC-151-44-s001.pdf]

Dietary fats and their sources in association with the risk of bladder cancer: a pooled analysis of 11 prospective cohort studies

Mostafa Dianatinasab, Anke Wesselius, Amin Salehi-Abargouei, Evan Y.W. Yu, Mohammad Fararouei, Maree Brinkman, Piet van den Brandt, Emily White, Elisabete Weiderpass, Florence Le Calvez-Kelm, Marc J. Gunter, Inge Huybrechts, Maurice P. Zeegers

Table of contents:

Supplementary Tables 1 - 3

Supplementary Table 1. Additional baseline characteristics for included studies in BLEND study

Characteristics	NLCS	VITAL	EPIC-Denmark	EPIC-France	EPIC-Germany	EPIC-Italy	EPIC-Spain	EPIC-Sweden	EPIC-the Netherlands	EPIC- the UK	EPIC-Norway
	N=5,238	N=66,518	N=55,670	N=64,204	N=48,754	N= 44,663	N=40,389	N=48,625	N=36,801	N= 74,379	N=33,304
**Stage**											
Non-invasive, CIS, Ta and T1	386	217	60	22	107	97	48	-	81	0	-
Muscle-invasive, T2-T4	413	104	22	4	36	19	7	-	22	4	-
**Initiate year of baseline assessment**	1986	2000	1993	1990	1994	1992	1992	1991	1993	1993	1991
**Length of follow up** (years)	14	7	11	10	10	11	12	13	12	11	10
**Subjects** (number)											
Case/	876/	337/	386/	31/	205/	186/	149/	301/	107/	247/	23/
non-case	4,362	66,181	55,284	64,173	48,549	44,477	40,240	48,324	36,694	74,132	33,281
**Person-year**	73688.8	448995.4	608813	667809.9	482453.3	502020.3	487491.1	638482.8	434974.5	828991.7	6437305.7
**Baseline age** (years) (mean ±SD)											
Case	62.73 (4.09)	66.16 (7.01)	58.50 (4.37)	58.04 (6.00)	56.41 (7.13)	55.24 (6.75)	54.49 (7.19)	60.27 (7.07)	56.20 (8.03)	63.62 (9.98)	49.30 (4.38)
Non-case	61.85 (4.21)	61.18 (7.37)	56.67 (4.37)	52.74 (6.63)	50.55 (8.56)	50.50 (7.92)	49.19 (8.03)	51.93 (10.89)	48.94 (11.93)	49.05 (14.34)	48.07 (4.30)
**Gender n (%)**					21,168 (43.42)	13,774 (30.84)	15,259 (37.78)	22,214 (45.68)	9,629 (26.17)	22,260 (29.93)	0 (0.00)
Men		33,394 (50.20)	26,532 (47.66)	0 (0.00)							33,304 (100.00)

Women	2,867 (54.73)	33,124 (49.80)	29,138 (52.34)	64,204 (100.00)	27,586 (56.58)	30,889 (69.16)	25,130 (62.22)	26,411 (54.32)	27,172 (73.83)	52,119 (70.07)
	2,371 (45.27)									

Abbreviations:

EPIC: European Prospective Investigation into Cancer

NLCS: The Netherlands Cohort Study

VITAL: VITamins and Lifestyle study.

Supplementary Table 2. Hazard ratio (HR) and 95% confidence interval (CI) of the association of oil and fat types, and risk of BC based on tertiles of intakes for stratification of gender.

	Tertile 1	Tertile 2	Tertile 3	*P-trend*		Tertile 1	Tertile 2	Tertile 3	*P-trend*
	HR (95%CI) *	HR (95%CI)	HR (95%CI)			HR (95%CI) *	HR (95%CI)	HR (95%CI)	
**Women**					**Men**				
Total lipid					***Total lipid***				
Case/non-case	144/ 102,374	159/ 103,923	154/ 97,467		Case/non-case	286/ 37,201	295/ 35,628	387/ 41,997	
Pearson year	1111433	1140187	1105761		Pearson year	411935.7	403310.4	506198.4	
Crude	1 (reference)	1.10 (0.88, 1.38)	0.98 (0.78, 1.24)	*0.911*	Crude	1 (reference)	1.12 (0.95, 1.33)	0.93 (0.80, 1.09)	*0.347*
Model 1 [1]	1 (reference)	1.11 (0.89, 1.40)	0.98 (0.77, 1.23)	*0.863*	Model 1 [1]	1 (reference)	1.15 (0.98, 1.36)	0.98 (0.83, 1.14)	*0.703*
Model 2 [2]	1 (reference)	1.13 (0.90, 1.42)	0.97 (0.77, 1.23)	*0.833*	Model 2 [2]	1 (reference)	1.15 (0.97, 1.36)	0.98 (0.83, 1.15)	*0.773*
Total Fatty acids					***Total Fatty acids***				
Case/non-case	133/ 105,134	154/ 106,809	170/ 91,821		Case/non-case	278/ 34,460	260/ 32,782	430/ 47,584	

Pearson year	1147538	1164204	1045638		Pearson year	391961.2	363196.5	566286.7	
Crude	1 (reference)	1.10 (0.87, 1.39)	1.23 (0.87, 1.39)	*0.069*	Crude	1 (reference)	0.93 (0.78, 1.10)	0.86 (0.73, 1.00)	*0.053*
Model 1 [1]	1 (reference)	1.04 (0.82, 1.31)	1.06 (0.84, 1.34)	*0.589*	Model 1 [1]	1 (reference)	0.93 (0.79, 1.11)	0.88 (0.76, 1.03)	*0.130*
Model 2 [2]	1 (reference)	1.03 (0.81, 1.31)	0.97 (0.76, 1.24)	*0.819*	Model 2 [2]	1 (reference)	0.96 (0.81, 1.15)	0.90 (0.76, 1.07)	*0.256*
Saturated fatty acids					***Saturated fatty acids***				
Case/non-case	143/ 106,587	126/ 101,948	188/ 95,229		Case/non-case	233/ 33,042	337/ 37,594	398/ 44,190	
Pearson year	1154378	1134166	1068837		Pearson year	369214.8	431727.2	520502.5	
Crude	1 (reference)	0.88 (0.69, 1.12)	1.21 (0.97, 1.51)	*0.067*	Crude	1 (reference)	1.26 (1.07, 1.49)	1.00 (0.85, 1.19)	*0.739*
Model 1 [1]	1 (reference)	0.89 (0.70, 1.13)	1.22 (0.98, 1.52)	*0.060*	Model 1 [1]	1 (reference)	1.29 (1.09, 1.52)	1.05 (0.89, 1.24)	*0.846*
Model 2 [2]	1 (reference)	0.87 (0.68, 1.11)	1.13 (0.90, 1.42)	*0.221*	Model 2 [2]	1 (reference)	1.28 (1.08, 1.51)	1.04 (0.88, 1.24)	*0.867*
Mono-unsaturated fatty acids					***Mono-unsaturated fatty acids***				
Case/non-case	166/ 98,563	147/ 102,840	144/ 102,361		Case/non-case	319/ 40,957	301/ 36,717	348/ 37,152	
Pearson year	1073998	1130764	1152618		Pearson year	452611.2	415495.3	453337.9	
Crude	1 (reference)	0.84 (0.67, 1.05)	0.71 (0.56, 0.89)	*0.003*	Crude	1 (reference)	1.04 (0.89, 1.22)	0.91 (0.78, 1.06)	*0.243*
Model 1 [1]	1 (reference)	0.80 (0.64, 1.00)	0.68 (0.54, 0.85)	*0.001*	Model 1 [1]	1 (reference)	1.05 (0.89, 1.23)	0.93 (0.79, 1.10)	*0.413*

Model 2 [2]	1 (reference)	0.86 (0.69, 1.08)	0.73 (0.58, 0.93)	*0.010*	Model 2 [2]	1 (reference)	1.06 (0.91, 1.25)	0.94 (0.80, 1.11)	*0.535*
Poly-unsaturated fatty acids					***Poly-unsaturated fatty acids***				
Case/non-case	166/ 108,103	132/ 104,099	159/ 91,562		Case/non-case	260/ 31,476	302/ 35,472	406/ 47,878	
Pearson year	1169728	1134201	1053452		Pearson year	347176.5	401709.9	572558	
Crude	1 (reference)	0.88 (0.70, 1.10)	1.12 (0.89, 1.40)	*0.341*	Crude	1 (reference)	1.09 (0.92, 1.29)	0.91 (0.78, 1.07)	*0.208*
Model 1 [1]	1 (reference)	0.85 (0.67, 1.07)	1.03 (0.82, 1.29)	*0.820*	Model 1 [1]	1 (reference)	1.10 (0.93, 1.30)	0.95 (0.81, 1.12)	*0.488*
Model 2 [2]	1 (reference)	0.86 (0.68, 1.08)	1.02 (0.81, 1.29)	*0.851*	Model 2 [2]	1 (reference)	1.09 (0.93, 1.29)	0.96 (0.82, 1.13)	*0.592*
Total Cholesterol					***Total Cholesterol***				
Case/non-case	152/ 98,433	156/ 101,110	149/ 104,221		Case/non-case	257/ 41,163	326/ 38,413	385/ 35,250	
Pearson year	1100883	1110559	1145938		Pearson year	481678.3	435062.5	404703.6	
Crude	1 (reference)	0.94 (0.75, 1.17)	0.84 (0.67, 1.06)	*0.150*	Crude	1 (reference)	1.25 (1.06, 1.47)	1.46 (1.25, 1.71)	*0.001*
Model 1 [1]	1 (reference)	0.94 (0.75, 1.18)	0.86 (0.68, 1.08)	*0.196*	Model 1 [1]	1 (reference)	1.18 (1.01, 1.40)	1.36 (1.16, 1.60)	*0.001*
Model 2 [2]	1 (reference)	0.96 (0.71, 1.13)	0.90 (0.71, 1.13)	*0.376*	Model 2 [2]	1 (reference)	1.19 (1.01, 1.41)	1.37 (1.16, 1.61)	*0.001*

◊ HR=Hazard Ratio, CI=confidence interval.

[1] adjusted for smoking status, age, and total energy intake in kilocalories.

[2] adjusted for model 1+ sugar and sugar products, beers, wine, dressing, vegetables and fruits.

Supplementary Table 3. Hazard ration (HR) and 95% confidence interval (CI) of the association of oil and fat types, and risk of BC based on tertiles of intakes for stratification of bladder cancer subtype.

	Tertile 1	Tertile 2	Tertile 3	*P-trend*		Tertile 1	Tertile 2	Tertile 3	*P-trend*
	HR (95%CI) *	HR (95%CI)	HR (95%CI)			HR (95%CI) *	HR (95%CI)	HR (95%CI)	
**NMIBC**					**MIBC**				
Total lipid					***Total lipid***				
Pearson year	1148.70	1209.08	654.63		Pearson year	281.15	366.05	158.14	
Crude	1 (reference)	0.90 (0.72, 1.12)	0.96 (0.73, 1.25)	*0.652*	Crude	1 (reference)	1.01 (0.66, 1.55)	0.78 (0.45, 1.34)	*0.437*
Model 1 [1]	1 (reference)	0.76 (0.60, 0.95)	0.75 (0.57, 0.98)	*0.020*	Model 1 [1]	1 (reference)	0.99 (0.63, 1.54)	1.14 (0.64, 2.00)	*0.701*
Model 2 [2]	1 (reference)	0.74 (0.60, 0.94)	0.73 (0.55, 0.96)	*0.014*	Model 2 [2]	1 (reference)	1.06 (0.67, 1.68)	1.19 (0.65, 2.17)	*0.569*
Total Fatty acids					***Total Fatty acids***				
Pearson year	1237.80	1066.81	707.81		Pearson year	230.76	296.60	277.98	
Crude	1 (reference)	0.96 (0.77, 1.21)	0.97 (0.75, 1.25)	*0.807*	Crude	1 (reference)	0.93 (0.58, 1.47)	0.61 (0.37, 1.00)	*0.047*
Model 1 [1]	1 (reference)	1.14 (0.89, 1.44)	1.11 (0.89, 1.44)	*0.379*	Model 1 [1]	1 (reference)	0.93 (0.58, 1.50)	0.66 (0.40, 1.10)	*0.111*
Model 2 [2]	1 (reference)	1.10 (0.85, 1.40)	1.01 (0.77, 1.32)	*0.864*	Model 2 [2]	1 (reference)	0.87 (0.52, 1.46)	0.58 (0.32, 1.05)	*0.065*

Saturated fatty acids					***Saturated fatty acids***				
Pearson year	946.77	1168.43	897.22		Pearson year	211.14	393.06	201.14	
Crude	1 (reference)	1.00 (0.79, 1.27)	1.04 (0.81, 1.33)	*0.745*	Crude	1 (reference)	0.96 (0.60, 1.51)	0.95 (0.57, 1.59)	*0.860*
Model 1 [1]	1 (reference)	0.83 (0.65, 1.05)	0.87 (0.67, 1.12)	*0.290*	Model 1 [1]	1 (reference)	0.95 (0.59, 1.55)	1.23 (0.71, 2.12)	*0.464*
Model 2 [2]	1 (reference)	0.84 (0.65, 1.07)	0.88 (0.68, 1.15)	*0.408*	Model 2 [2]	1 (reference)	0.88 (0.53, 1.45)	1.35 (0.76, 2.38)	*0.366*
Mono-unsaturated fatty acids					***Mono-unsaturated fatty acids***				
Pearson year	1290.45	1045.06	676.91		Pearson year	306.39	368.55	130.39	
Crude	1 (reference)	0.87 (0.70, 1.09)	0.88 (0.68, 1.14)	*0.273*	Crude	1 (reference)	0.82 (0.54, 1.25)	0.63 (0.36, 1.11)	*0.107*
Model 1 [1]	1 (reference)	0.71 (0.57, 0.90)	0.68 (0.52, 0.89)	*0.002*	Model 1 [1]	1 (reference)	0.87 (0.70, 1.09)	0.88 (0.68, 1.14)	*0.050*
Model 2 [2]	1 (reference)	0.74 (0.59, 0.94)	0.69 (0.53, 0.91)	*0.004*	Model 2 [2]	1 (reference)	0.75 (0.47, 1.18)	0.86 (0.44, 1.64)	*0.436*
Poly-unsaturated fatty acids					***Poly-unsaturated fatty acids***				
Pearson year	1188.09	1328.95	495.38		Pearson year	295.86	318.87	190.61	
Crude	1 (reference)	1.02 (0.82, 1.27)	1.04 (0.78, 1.38)	*0.745*	Crude	1 (reference)	1.10 (0.71, 1.70)	0.90 (0.54, 1.50)	*0.795*
Model 1 [1]	1 (reference)	0.85 (0.68, 1.06)	0.89 (0.67, 1.19)	*0.304*	Model 1 [1]	1 (reference)	1.12 (0.71, 1.76)	1.18 (0.70, 1.98)	*0.497*

Model 2 [2]	1 (reference)	0.86 (0.69, 1.08)	0.85 (0.63, 1.15)	*0.211*	Model 2 [2]	1 (reference)	1.09 (0.69, 1.74)	1.16 (0.67, 1.98)	*0.574*
Total Cholesterol					***Total Cholesterol***				
Pearson year	776.76	1027.05	1208.617		Pearson year	173.96	354.93	276.44	
Crude	1 (reference)	1.24 (0.96, 1.61)	1.12 (0.87, 1.44)	*0.447*	Crude	1 (reference)	1.16 (0.69, 1.95)	1.22 (0.72, 2.08)	*0.431*
Model 1 [1]	1 (reference)	1.28 (0.98, 1.66)	0.90 (0.70, 1.16)	*0.251*	Model 1 [1]	1 (reference)	1.33 (0.77, 2.28)	1.19 (0.69, 2.06)	*0.611*
Model 2 [2]	1 (reference)	1.29 (0.99, 1.69)	1.01 (0.77, 1.31)	*0.825*	Model 2 [2]	1 (reference)	1.27 (0.73, 2.21)	1.14 (0.63, 2.07)	*0.736*

[◊] HR=Hazard Ratio, CI=confidence interval.

[1] adjusted for smoking status, age, gender and total energy intake in kilocalories.

[2] adjusted for model 1+ sugar and sugar products, beers, wine, dressing, vegetables and fruits.
